# 

**Published:** 1887-05

**Authors:** 


COLLABORATORS.

CHICAGO:

PROFESSOR E. ANDREWS, M.D., LL.D.
PROFESSOR J. BARTLETT, M.D.
PROFESSOR W. H. BYFORD, A.M., M.D.
PROFESSOR L. CURTIS, A.M., M.D.
PROFESSOR O. C. DEWOLF, A M., M.D.
PROFESSOR E. C. DUDLEY, A.B., M.D.
PROFESSOR C. FENGER, M.D.
PROFESSOR ELBERT WING, A.M., M.D.
PROFESSOR J. N. HYDE, A.M., M.D.
PROFESSOR E. F. INGALS, A.M., M D.
PROFESSOR W. W. JAGGARD, A.M., M.D.
PROFESSOR F. S. JOHNSON, A.M., M.D.
PROFESSOR H. A. JOHNSON, M.D., LL.D.
PROFESSOR C. W. PURDY, M.D.
PROFESSOR C. G. SMITH, A.M., M.D.
PROFESSOR F. BILLINGS, M.D.
PROFESSOR W. T. BELFIELD, M.D.

MILWAUKEE, WISCONSIN:

PROFESSOR N. SENN, M.D.

WASHINGTON, D. C.:

S. O. RICHEY, M.D.

UNITED STATES ARMY:

SURGEON D. L. HUNTINGTON, M.D.

UNITED STATES NAVY

MEDICAL DIRECTOR J. M. BROWNE, M.D.
MEDICAL DIRECTOR A. L. GIHON, A.M., M.D.

